# Bis[diamino­(ethoxy­carbonyl­amino)­methyl­ium] sulfate

**DOI:** 10.1107/S160053680900912X

**Published:** 2009-03-19

**Authors:** M. Nawaz Tahir, Christy Muir, Muhammad Danish, Muhammad Ilyas Tariq, Dinçer Ülkü

**Affiliations:** a University of Sargodha, Department of Physics, Sagrodha, Pakistan; b Department of Chemistry, FC College University, Lahore, Pakistan; c University of Sargodha, Department of Chemistry, Sagrodha, Pakistan; d Hacettepe University, Department of Physics Engineering, Beytepe 06532, Ankara, Turkey

## Abstract

In the mol­ecule of the title compound, 2C_4_H_10_N_3_O_2_^+^·SO_4_^−^, the cations are planar (r.m.s. deviations = 0.0144 and 0.0236 Å) and oriented at a dihedral angle of 62.30 (4)°. Intra­molecular N—H⋯O hydrogen bonds result in the formation of two planar six-membered rings. The cations are linked to the sulfate ion through inter­molecular C—H⋯O and N—H⋯O hydrogen bonds, forming an *R*_2_^2^(8) ring motif. In the crystal structure, inter­molecular N—H⋯O and C—H⋯O hydrogen bonds link the mol­ecules into a three-dimensional network.

## Related literature

For related structures, see: Brauer & Kottsieper (2003 ▶); Curtis & Pasternak (1955 ▶). For bond-length data, see: Allen *et al.* (1987 ▶). For ring motifs, see: Bernstein *et al.* (1995 ▶).
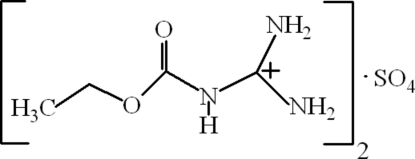


## Experimental

### 

#### Crystal data

2C_4_H_10_N_3_O_2_^+^·SO_4_^2−^
*M* *_r_* = 360.36
Monoclinic, 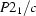

*a* = 9.3021 (12) Å
*b* = 11.0081 (11) Å
*c* = 17.1063 (13) Å
β = 100.980 (3)°
*V* = 1719.6 (3) Å^3^
*Z* = 4
Mo *K*α radiation
μ = 0.24 mm^−1^
*T* = 296 K
0.24 × 0.18 × 0.15 mm

#### Data collection

Enraf–Nonius CAD-4 diffractometer
Absorption correction: ψ scan (North *et al.*, 1968 ▶) *T*_min_ = 0.946, *T*_max_ = 0.967
3481 measured reflections
3481 independent reflections
2124 reflections with *I* > 2σ(*I*)
*R*_int_ = 0.025
3 standard reflections frequency: 120 min intensity decay: 1.7%

#### Refinement

*R*[*F* ^2^ > 2σ(*F* ^2^)] = 0.045
*wR*(*F* ^2^) = 0.108
*S* = 1.03
3481 reflections
233 parameters
H atoms treated by a mixture of independent and constrained refinement
Δρ_max_ = 0.21 e Å^−3^
Δρ_min_ = −0.25 e Å^−3^

### 

Data collection: *CAD-4 EXPRESS* (Enraf–Nonius, 1994 ▶); cell refinement: *CAD-4 EXPRESS*; data reduction: *XCAD4* (Harms & Wocadlo, 1995 ▶); program(s) used to solve structure: *SHELXS97* (Sheldrick, 2008 ▶); program(s) used to refine structure: *SHELXL97* (Sheldrick, 2008 ▶); molecular graphics: *ORTEP-3 for Windows* (Farrugia, 1997 ▶) and *PLATON* (Spek, 2009 ▶); software used to prepare material for publication: *WinGX* (Farrugia, 1999 ▶) and *PLATON* (Spek, 2009 ▶).

## Supplementary Material

Crystal structure: contains datablocks global, I. DOI: 10.1107/S160053680900912X/hk2641sup1.cif

Structure factors: contains datablocks I. DOI: 10.1107/S160053680900912X/hk2641Isup2.hkl

Additional supplementary materials:  crystallographic information; 3D view; checkCIF report

## Figures and Tables

**Table 1:** Hydrogen-bond geometry (Å, °)

*D*—H⋯*A*	*D*—H	H⋯*A*	*D*⋯*A*	*D*—H⋯*A*
N1—H1*A*⋯O1^i^	0.95 (3)	1.89 (3)	2.837 (3)	175 (3)
N1—H1*B*⋯O2^ii^	0.84 (3)	1.96 (3)	2.805 (3)	177 (3)
N2—H2*A*⋯O5	0.82 (3)	2.10 (3)	2.712 (3)	131 (3)
N2—H2*A*⋯O7^iii^	0.82 (3)	2.27 (3)	2.975 (3)	144 (3)
N2—H2*B*⋯O1^ii^	0.90 (3)	1.95 (3)	2.854 (3)	174 (3)
N4—H4*D*⋯O3^i^	0.91 (3)	2.00 (3)	2.841 (3)	153 (2)
N4—H4*E*⋯O2	0.91 (3)	1.90 (3)	2.813 (3)	176 (3)
N3—H5⋯O4^i^	0.86	1.94	2.769 (3)	163
N5—H5*A*⋯O7	0.81 (3)	2.14 (3)	2.730 (3)	130 (3)
N5—H5*A*⋯O5^iv^	0.81 (3)	2.32 (3)	3.031 (3)	147 (3)
N5—H5*B*⋯O4	0.93 (3)	1.97 (3)	2.898 (3)	177 (3)
N6—H6⋯O3^i^	0.86	1.95	2.752 (3)	155
C3—H3*A*⋯O3	0.97	2.58	3.368 (4)	138
C7—H7*B*⋯O2^v^	0.97	2.55	3.483 (3)	162

Symmetry codes: (i) 
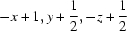
; (ii) 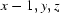; (iii) 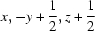; (iv) 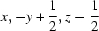; (v) 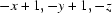.

**Fig. 1.**
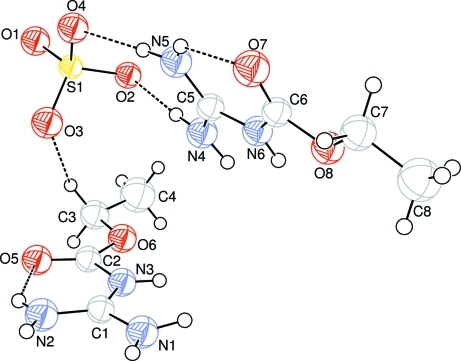
The molecular structure of the title molecule, with the atom-numbering scheme. Hydrogen bonds are shown as dashed lines.

**Fig. 2.**
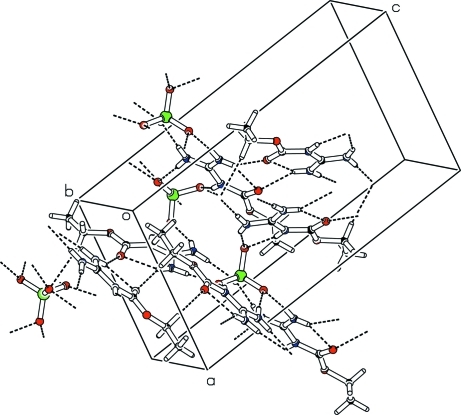
A partial packing diagram of the title compound. Hydrogen bonds are shown as dashed lines.

## supplementary crystallographic information

### Comment

As part of our ongoing studies, we report herein the crystal structure of the title compound, (I).

The crystal structures of 1-carbamoylguanidinium methylphosphonate monohydrate, (II) (Brauer & Kottsieper, 2003) and methylguanidinium nitrate, (III) (Curtis & Pasternak, 1955) have been reported. In the molecule of the title compound (Fig. 1), the bond lengths (Allen *et al.*,  1987) and angles of the diamino(ethoxycarbonylamino)methylium (DEAM) moieties are within normal ranges. DEAM moieties (N1–N3/O5/O6/C1–C4) and (N4–N6/O7/O8/C5–C8) are planar with maximum deviations of 0.043 (2) and -0.322 (3) Å for N2 and N4 atoms, respectively, in which they are oriented at a dihedral angle of 62.30 (4)°. The intramolecular N—H···O hydrogen bonds result in the formations of two planar six-membered rings: A (O5/N2/N3/C1/C2/H2A) and B (O7/N5/N6/C5/C6/H5A). The dihedral angle between them is A/B = 60.38 (3)°. The DEAM moieties are linked to the SO_4_ ion through the intramolecular C—H···O and N—H···O hydrogen bonds (Table 1), forming a R_2_^2^(8) ring motif (Bernstein *et al.*,  1995).

In the crystal structure, intermolecular N—H···O and C—H···O hydrogen bonds (Table 1) link the molecules into a three dimensional network (Fig. 2), in which they may be effective in the stabilization of the structure.

### Experimental

For the preparation of the title compound, 1-cyanoguanidine (2.1 g, 0.025 mol) was dissolved in water (50 ml), and then a few drops of H_2_SO_4_ were added. The resulting mixture was refluxed for 2–3 h, and cooled to room temperature. The excess of ethanol was added, and then refluxed for 2–3 h. It was filtered through alumina. The filtrate was concentrated under reduced pressure and kept for crystallization. Recrystallization was carried out from ethanol/hexane (9:1) mixture in 5 d.

### Crystal data

**Table:**

2C_4_H_10_N_3_O_2_^+^·SO_4_^2^^−^	*F*(000) = 760
*M**_r_* = 360.36	*D*_x_ = 1.392 Mg m^−^^3^
Monoclinic, *P*2_1_/*c*	Mo *K*α radiation, λ = 0.71073 Å
Hall symbol: -P 2ybc	Cell parameters from 25 reflections
*a* = 9.3021 (12) Å	θ = 10.0–18.2°
*b* = 11.0081 (11) Å	µ = 0.24 mm^−^^1^
*c* = 17.1063 (13) Å	*T* = 296 K
β = 100.980 (3)°	Prism, colourless
*V* = 1719.6 (3) Å^3^	0.24 × 0.18 × 0.15 mm
*Z* = 4	

### Data collection

**Table:**

Enraf–Nonius CAD-4 diffractometer	2124 reflections with *I* > 2σ(*I*)
Radiation source: fine-focus sealed tube	*R*_int_ = 0.025
graphite	θ_max_ = 26.3°, θ_min_ = 2.2°
ω/2θ scans	*h* = 0→11
Absorption correction: ψ scan (North *et al.*, 1968)	*k* = 0→13
*T*_min_ = 0.946, *T*_max_ = 0.967	*l* = −21→20
3481 measured reflections	3 standard reflections every 120 min
3481 independent reflections	intensity decay: 1.7%

### Refinement

**Table:**

Refinement on *F*^2^	Secondary atom site location: difference Fourier map
Least-squares matrix: full	Hydrogen site location: inferred from neighbouring sites
*R*[*F*^2^ > 2σ(*F*^2^)] = 0.045	H atoms treated by a mixture of independent and constrained refinement
*wR*(*F*^2^) = 0.108	*w* = 1/[σ^2^(*F*_o_^2^) + (0.0481*P*)^2^ + 0.1093*P*] where *P* = (*F*_o_^2^ + 2*F*_c_^2^)/3
*S* = 1.03	(Δ/σ)_max_ < 0.001
3481 reflections	Δρ_max_ = 0.21 e Å^−^^3^
233 parameters	Δρ_min_ = −0.25 e Å^−^^3^
0 restraints	Extinction correction: *SHELXL97* (Sheldrick, 2008), Fc^*^=kFc[1+0.001xFc^2^λ^3^/sin(2θ)]^-1/4^
Primary atom site location: structure-invariant direct methods	Extinction coefficient: 0.0037 (9)

### Special details

**Table:**

Geometry. Bond distances, angles *etc*. have been calculated using the rounded fractional coordinates. All su's are estimated from the variances of the (full) variance-covariance matrix. The cell e.s.d.'s are taken into account in the estimation of distances, angles and torsion angles
Refinement. Refinement of *F*^2^ against ALL reflections. The weighted *R*-factor *wR* and goodness of fit *S* are based on *F*^2^, conventional *R*-factors *R* are based on *F*, with *F* set to zero for negative *F*^2^. The threshold expression of *F*^2^ > σ(*F*^2^) is used only for calculating *R*-factors(gt) *etc*. and is not relevant to the choice of reflections for refinement. *R*-factors based on *F*^2^ are statistically about twice as large as those based on *F*, and *R*- factors based on ALL data will be even larger.

### Fractional atomic coordinates and isotropic or equivalent isotropic displacement parameters (Å^2^)

**Table:**

	*x*	*y*	*z*	*U*_iso_*/*U*_eq_	
S1	0.74538 (6)	0.22561 (5)	0.25630 (4)	0.0324 (2)	
O1	0.87642 (18)	0.15672 (16)	0.29258 (11)	0.0494 (6)	
O2	0.78056 (17)	0.35534 (15)	0.25361 (10)	0.0398 (6)	
O3	0.6301 (2)	0.20989 (17)	0.30259 (11)	0.0536 (7)	
O4	0.6925 (2)	0.18149 (16)	0.17468 (10)	0.0492 (7)	
O5	0.43306 (19)	0.28294 (17)	0.43071 (11)	0.0499 (6)	
O6	0.53979 (17)	0.45797 (16)	0.40549 (11)	0.0471 (6)	
O7	0.2706 (2)	0.42933 (17)	−0.03291 (11)	0.0560 (7)	
O8	0.17269 (19)	0.60725 (16)	−0.00697 (10)	0.0460 (6)	
N1	0.0671 (3)	0.4461 (2)	0.29228 (14)	0.0499 (8)	
N2	0.1472 (3)	0.2719 (2)	0.35872 (15)	0.0529 (9)	
N3	0.3058 (2)	0.43420 (19)	0.35471 (12)	0.0422 (7)	
N4	0.5287 (3)	0.4909 (2)	0.19080 (14)	0.0500 (8)	
N5	0.4929 (3)	0.3569 (2)	0.08613 (14)	0.0480 (8)	
N6	0.3530 (2)	0.53306 (19)	0.08234 (12)	0.0426 (7)	
C1	0.1711 (3)	0.3811 (2)	0.33493 (15)	0.0382 (8)	
C2	0.4290 (3)	0.3811 (2)	0.40006 (15)	0.0381 (8)	
C3	0.6785 (3)	0.4149 (3)	0.45146 (17)	0.0532 (10)	
C4	0.7871 (3)	0.5128 (3)	0.4471 (2)	0.0807 (15)	
C5	0.4611 (3)	0.4578 (2)	0.11953 (15)	0.0376 (8)	
C6	0.2642 (3)	0.5151 (3)	0.00936 (15)	0.0387 (8)	
C7	0.0712 (3)	0.6005 (3)	−0.08355 (15)	0.0502 (10)	
C8	−0.0188 (4)	0.7126 (3)	−0.0906 (2)	0.0738 (12)	
H1A	0.090 (3)	0.518 (3)	0.2665 (16)	0.0599*	
H1B	−0.020 (3)	0.421 (3)	0.2797 (17)	0.0599*	
H2A	0.215 (3)	0.234 (3)	0.3859 (18)	0.0634*	
H2B	0.059 (3)	0.237 (3)	0.3410 (17)	0.0634*	
H3A	0.70789	0.34004	0.42905	0.0638*	
H3B	0.67011	0.40013	0.50630	0.0638*	
H4A	0.88070	0.48943	0.47744	0.0965*	
H4B	0.75542	0.58660	0.46846	0.0965*	
H4C	0.79518	0.52545	0.39258	0.0965*	
H4D	0.499 (3)	0.563 (3)	0.2083 (16)	0.0600*	
H4E	0.608 (3)	0.445 (3)	0.2123 (16)	0.0600*	
H5	0.31494	0.50670	0.33757	0.0505*	
H5A	0.446 (3)	0.337 (3)	0.0435 (17)	0.0576*	
H5B	0.560 (3)	0.303 (3)	0.1145 (16)	0.0576*	
H6	0.33899	0.59866	0.10715	0.0511*	
H7A	0.00927	0.52921	−0.08540	0.0602*	
H7B	0.12481	0.59560	−0.12684	0.0602*	
H8A	−0.09353	0.70817	−0.13771	0.0883*	
H8B	0.04250	0.78183	−0.09397	0.0883*	
H8C	−0.06354	0.72035	−0.04471	0.0883*	

### Atomic displacement parameters (Å^2^)

**Table:**

	*U*^11^	*U*^22^	*U*^33^	*U*^12^	*U*^13^	*U*^23^
S1	0.0322 (3)	0.0241 (3)	0.0383 (4)	−0.0023 (3)	−0.0001 (3)	0.0012 (3)
O1	0.0432 (11)	0.0334 (10)	0.0616 (12)	0.0064 (9)	−0.0152 (9)	−0.0021 (9)
O2	0.0347 (9)	0.0263 (9)	0.0566 (11)	−0.0042 (7)	0.0041 (8)	0.0042 (8)
O3	0.0547 (12)	0.0461 (12)	0.0647 (12)	−0.0088 (10)	0.0234 (10)	0.0106 (10)
O4	0.0594 (12)	0.0378 (11)	0.0425 (11)	0.0086 (9)	−0.0106 (9)	−0.0060 (8)
O5	0.0432 (10)	0.0396 (11)	0.0625 (12)	−0.0056 (9)	−0.0011 (9)	0.0182 (10)
O6	0.0315 (9)	0.0409 (11)	0.0639 (12)	−0.0073 (8)	−0.0032 (8)	0.0060 (9)
O7	0.0609 (12)	0.0462 (12)	0.0535 (12)	0.0160 (10)	−0.0080 (9)	−0.0187 (10)
O8	0.0512 (11)	0.0440 (11)	0.0394 (10)	0.0178 (9)	0.0002 (8)	−0.0017 (9)
N1	0.0359 (12)	0.0444 (15)	0.0620 (16)	−0.0101 (12)	−0.0097 (12)	0.0166 (12)
N2	0.0430 (14)	0.0397 (15)	0.0673 (16)	−0.0156 (12)	−0.0113 (12)	0.0165 (12)
N3	0.0344 (11)	0.0336 (13)	0.0537 (13)	−0.0085 (10)	−0.0036 (10)	0.0124 (10)
N4	0.0540 (15)	0.0426 (15)	0.0466 (14)	0.0216 (12)	−0.0074 (11)	−0.0097 (12)
N5	0.0548 (15)	0.0379 (14)	0.0451 (14)	0.0159 (12)	−0.0062 (11)	−0.0088 (12)
N6	0.0498 (13)	0.0340 (12)	0.0408 (12)	0.0139 (11)	0.0006 (10)	−0.0078 (10)
C1	0.0365 (14)	0.0356 (16)	0.0394 (14)	−0.0084 (12)	−0.0004 (11)	0.0031 (12)
C2	0.0367 (14)	0.0365 (16)	0.0394 (14)	−0.0071 (12)	0.0033 (11)	0.0010 (12)
C3	0.0355 (15)	0.0536 (19)	0.0644 (19)	−0.0006 (14)	−0.0058 (13)	−0.0074 (15)
C4	0.0395 (17)	0.093 (3)	0.105 (3)	−0.0204 (18)	0.0024 (17)	−0.012 (2)
C5	0.0391 (14)	0.0342 (15)	0.0388 (15)	0.0066 (12)	0.0055 (12)	−0.0001 (12)
C6	0.0396 (14)	0.0345 (15)	0.0417 (15)	0.0051 (12)	0.0069 (12)	−0.0004 (13)
C7	0.0500 (16)	0.0579 (19)	0.0384 (15)	0.0098 (15)	−0.0022 (12)	0.0020 (14)
C8	0.070 (2)	0.075 (2)	0.070 (2)	0.032 (2)	−0.0028 (17)	0.0115 (19)

### Geometric parameters (Å, °)

**Table:**

S1—O3	1.460 (2)	N5—C5	1.308 (3)
S1—O4	1.4716 (18)	N6—C5	1.363 (3)
S1—O1	1.4690 (19)	N6—C6	1.374 (3)
S1—O2	1.4678 (17)	N4—H4E	0.91 (3)
O5—C2	1.199 (3)	N4—H4D	0.91 (3)
O6—C3	1.457 (3)	N5—H5B	0.93 (3)
O6—C2	1.323 (3)	N5—H5A	0.81 (3)
O7—C6	1.198 (4)	N6—H6	0.8600
O8—C6	1.320 (4)	C3—C4	1.489 (4)
O8—C7	1.464 (3)	C3—H3A	0.9700
N1—C1	1.308 (4)	C3—H3B	0.9700
N2—C1	1.302 (3)	C4—H4C	0.9600
N3—C2	1.385 (3)	C4—H4B	0.9600
N3—C1	1.366 (3)	C4—H4A	0.9600
N1—H1A	0.95 (3)	C7—C8	1.483 (5)
N1—H1B	0.84 (3)	C7—H7A	0.9700
N2—H2A	0.82 (3)	C7—H7B	0.9700
N2—H2B	0.90 (3)	C8—H8B	0.9600
N3—H5	0.8600	C8—H8C	0.9600
N4—C5	1.312 (3)	C8—H8A	0.9600
			
S1···H2B^i^	3.00 (3)	N4···O2	2.813 (3)
S1···H4E	2.77 (3)	N5···O4	2.898 (3)
S1···H5B	2.83 (3)	N5···O7	2.730 (3)
S1···H1A^ii^	2.82 (3)	N5···C6^v^	3.341 (4)
S1···H4D^ii^	3.04 (3)	N5···O5^iv^	3.031 (3)
S1···H5^ii^	2.8900	N6···O3^vii^	2.752 (3)
S1···H6^ii^	2.9500	C2···O3	3.320 (3)
S1···H1B^i^	3.04 (3)	C3···O3	3.368 (4)
O1···N2^i^	2.854 (3)	C5···O3^vii^	3.260 (3)
O1···N1^ii^	2.837 (3)	C5···O7^v^	3.373 (3)
O2···N1^i^	2.805 (3)	C6···N5^v^	3.341 (4)
O2···N4	2.813 (3)	C2···H4B^viii^	3.1000
O3···C2	3.320 (3)	C2···H2A	2.54 (3)
O3···N4^ii^	2.841 (3)	C6···H5A	2.58 (3)
O3···C5^ii^	3.260 (3)	H1A···S1^vii^	2.82 (3)
O3···C3	3.368 (4)	H1A···O1^vii^	1.89 (3)
O3···O5	3.216 (3)	H1A···H5	2.2100
O3···N6^ii^	2.752 (3)	H1A···O4^vii^	2.75 (3)
O4···N5	2.898 (3)	H1B···S1^vi^	3.04 (3)
O4···N3^ii^	2.769 (3)	H1B···H2B	2.33 (5)
O5···O7^iii^	2.914 (3)	H1B···O2^vi^	1.96 (3)
O5···O3	3.216 (3)	H2A···O7^iii^	2.27 (3)
O5···N2	2.712 (3)	H2A···C2	2.54 (3)
O5···N5^iii^	3.031 (3)	H2A···O5	2.10 (3)
O7···O5^iv^	2.914 (3)	H2B···O1^vi^	1.95 (3)
O7···N5	2.730 (3)	H2B···H1B	2.33 (5)
O7···N2^iv^	2.975 (3)	H2B···S1^vi^	3.00 (3)
O7···C5^v^	3.373 (3)	H3A···O5	2.6400
O1···H2B^i^	1.95 (3)	H3A···O3	2.5800
O1···H1A^ii^	1.89 (3)	H3B···O5	2.6700
O2···H1B^i^	1.96 (3)	H4A···H4A^ix^	2.2200
O2···H5B	2.89 (3)	H4B···C2^viii^	3.1000
O2···H4E	1.90 (3)	H4D···S1^vii^	3.04 (3)
O2···H7B^v^	2.5500	H4D···H6	2.0900
O3···H3A	2.5800	H4D···O3^vii^	2.00 (3)
O3···H4D^ii^	2.00 (3)	H4E···S1	2.77 (3)
O3···H6^ii^	1.9500	H4E···H5B	2.27 (4)
O4···H5B	1.97 (3)	H4E···O2	1.90 (3)
O4···H1A^ii^	2.75 (3)	H5···H1A	2.2100
O4···H5^ii^	1.9400	H5···S1^vii^	2.8900
O5···H3A	2.6400	H5···O4^vii^	1.9400
O5···H3B	2.6700	H5A···C6	2.58 (3)
O5···H5A^iii^	2.32 (3)	H5A···O5^iv^	2.32 (3)
O5···H2A	2.10 (3)	H5A···O7	2.14 (3)
O7···H7B	2.6300	H5B···O2	2.89 (3)
O7···H7A	2.6600	H5B···O4	1.97 (3)
O7···H5A	2.14 (3)	H5B···S1	2.83 (3)
O7···H2A^iv^	2.27 (3)	H5B···H4E	2.27 (4)
N1···O2^vi^	2.805 (3)	H6···S1^vii^	2.9500
N1···O1^vii^	2.837 (3)	H6···O3^vii^	1.9500
N2···O1^vi^	2.854 (3)	H6···H4D	2.0900
N2···O5	2.712 (3)	H7A···O7	2.6600
N2···O7^iii^	2.975 (3)	H7B···O7	2.6300
N3···O4^vii^	2.769 (3)	H7B···O2^v^	2.5500
N4···O3^vii^	2.841 (3)		
			
O3—S1—O4	109.16 (11)	O5—C2—N3	125.4 (2)
O1—S1—O3	110.23 (11)	O6—C3—C4	106.1 (2)
O1—S1—O4	109.33 (11)	H3A—C3—H3B	109.00
O1—S1—O2	110.09 (10)	O6—C3—H3B	111.00
O2—S1—O4	109.12 (10)	O6—C3—H3A	111.00
O2—S1—O3	108.88 (10)	C4—C3—H3A	111.00
C2—O6—C3	115.3 (2)	C4—C3—H3B	111.00
C6—O8—C7	115.5 (2)	C3—C4—H4C	109.00
C1—N3—C2	125.5 (2)	H4A—C4—H4B	109.00
C1—N1—H1A	120.5 (17)	H4A—C4—H4C	109.00
C1—N1—H1B	122 (2)	C3—C4—H4A	109.00
H1A—N1—H1B	116 (3)	C3—C4—H4B	109.00
C1—N2—H2A	119 (2)	H4B—C4—H4C	109.00
C1—N2—H2B	119 (2)	N4—C5—N5	122.2 (2)
H2A—N2—H2B	122 (3)	N4—C5—N6	116.4 (2)
C2—N3—H5	117.00	N5—C5—N6	121.3 (2)
C1—N3—H5	117.00	O7—C6—N6	124.9 (3)
C5—N6—C6	126.8 (2)	O7—C6—O8	125.6 (2)
C5—N4—H4E	115.4 (18)	O8—C6—N6	109.5 (2)
H4D—N4—H4E	129 (3)	O8—C7—C8	106.8 (2)
C5—N4—H4D	115.2 (17)	C8—C7—H7A	110.00
C5—N5—H5B	120.0 (18)	C8—C7—H7B	110.00
C5—N5—H5A	120 (2)	O8—C7—H7A	110.00
H5A—N5—H5B	120 (3)	O8—C7—H7B	110.00
C6—N6—H6	117.00	H7A—C7—H7B	109.00
C5—N6—H6	117.00	H8B—C8—H8C	109.00
N2—C1—N3	121.4 (2)	C7—C8—H8A	109.00
N1—C1—N3	116.8 (2)	C7—C8—H8B	109.00
N1—C1—N2	121.8 (3)	C7—C8—H8C	109.00
O6—C2—N3	108.65 (19)	H8A—C8—H8B	109.00
O5—C2—O6	125.9 (2)	H8A—C8—H8C	109.00
			
C3—O6—C2—O5	1.9 (4)	C2—N3—C1—N2	0.4 (4)
C3—O6—C2—N3	−179.8 (2)	C1—N3—C2—O5	−3.0 (4)
C2—O6—C3—C4	178.0 (2)	C1—N3—C2—O6	178.6 (2)
C6—O8—C7—C8	−179.0 (2)	C6—N6—C5—N4	−177.4 (3)
C7—O8—C6—O7	0.5 (4)	C6—N6—C5—N5	1.2 (4)
C7—O8—C6—N6	179.8 (2)	C5—N6—C6—O7	−0.7 (4)
C2—N3—C1—N1	179.2 (2)	C5—N6—C6—O8	180.0 (2)

Symmetry codes: (i) *x*+1, *y*, *z*; (ii) −*x*+1, *y*−1/2, −*z*+1/2; (iii) *x*, −*y*+1/2, *z*+1/2; (iv) *x*, −*y*+1/2, *z*−1/2; (v) −*x*+1, −*y*+1, −*z*; (vi) *x*−1, *y*, *z*; (vii) −*x*+1, *y*+1/2, −*z*+1/2; (viii) −*x*+1, −*y*+1, −*z*+1; (ix) −*x*+2, −*y*+1, −*z*+1.

### Hydrogen-bond geometry (Å, °)

**Table:**

*D*—H···*A*	*D*—H	H···*A*	*D*···*A*	*D*—H···*A*
N1—H1A···O1^vii^	0.95 (3)	1.89 (3)	2.837 (3)	175 (3)
N1—H1B···O2^vi^	0.84 (3)	1.96 (3)	2.805 (3)	177 (3)
N2—H2A···O5	0.82 (3)	2.10 (3)	2.712 (3)	131 (3)
N2—H2A···O7^iii^	0.82 (3)	2.27 (3)	2.975 (3)	144 (3)
N2—H2B···O1^vi^	0.90 (3)	1.95 (3)	2.854 (3)	174 (3)
N4—H4D···O3^vii^	0.91 (3)	2.00 (3)	2.841 (3)	153 (2)
N4—H4E···O2	0.91 (3)	1.90 (3)	2.813 (3)	176 (3)
N3—H5···O4^vii^	0.86	1.94	2.769 (3)	163
N5—H5A···O7	0.81 (3)	2.14 (3)	2.730 (3)	130 (3)
N5—H5A···O5^iv^	0.81 (3)	2.32 (3)	3.031 (3)	147 (3)
N5—H5B···O4	0.93 (3)	1.97 (3)	2.898 (3)	177 (3)
N6—H6···O3^vii^	0.86	1.95	2.752 (3)	155
C3—H3A···O3	0.97	2.58	3.368 (4)	138
C7—H7B···O2^v^	0.97	2.55	3.483 (3)	162

Symmetry codes: (vii) −*x*+1, *y*+1/2, −*z*+1/2; (vi) *x*−1, *y*, *z*; (iii) *x*, −*y*+1/2, *z*+1/2; (iv) *x*, −*y*+1/2, *z*−1/2; (v) −*x*+1, −*y*+1, −*z*.

## References

[bb1] Allen, F. H., Kennard, O., Watson, D. G., Brammer, L., Orpen, A. G. & Taylor, R. (1987). *J. Chem. Soc. Perkin Trans. 2*, pp. S1–19.

[bb2] Bernstein, J., Davis, R. E., Shimoni, L. & Chang, N.-L. (1995). *Angew. Chem. Int. Ed. Engl.* **34**, 1555–1573.

[bb3] Brauer, D. J. & Kottsieper, K. W. (2003). *Acta Cryst.* C**59**, o244–o246. 10.1107/s0108270103005857 12743404

[bb4] Curtis, R. M. & Pasternak, R. A. (1955). *Acta Cryst.* **8**, 675–681.

[bb5] Enraf–Nonius (1994). *CAD-4 EXPRESS* Enraf–Nonius, Delft, The Netherlands.

[bb6] Farrugia, L. J. (1997). *J. Appl. Cryst.* **30**, 565.

[bb7] Farrugia, L. J. (1999). *J. Appl. Cryst.* **32**, 837–838.

[bb8] Harms, K. & Wocadlo, S. (1995). *XCAD4* University of Marburg, Germany.

[bb9] North, A. C. T., Phillips, D. C. & Mathews, F. S. (1968). *Acta Cryst.* A**24**, 351–359.

[bb10] Sheldrick, G. M. (2008). *Acta Cryst.* A**64**, 112–122. 10.1107/S0108767307043930 18156677

[bb11] Spek, A. L. (2009). *Acta Cryst.* D**65**, 148–155. 10.1107/S090744490804362X PMC2631630 19171970

